# Australian national birthweight percentiles by sex and gestational age for twins, 2001–2010

**DOI:** 10.1186/s12887-015-0464-y

**Published:** 2015-10-08

**Authors:** Zhuoyang Li, Mark P. Umstad, Lisa Hilder, Fenglian Xu, Elizabeth A. Sullivan

**Affiliations:** Faculty of Health, University of Technology Sydney, Sydney, Australia; National Perinatal Epidemiology and Statistics Units, University of New South Wales, Sydney, Australia; The Royal Women’s Hospital, Melbourne, Australia; The University of Melbourne Department of Obstetrics and Gynaecology, Melbourne, Australia

**Keywords:** Twins, Birth weight, Gestational age, Small for gestational age

## Abstract

**Background:**

Birthweight remains one of the strongest predictors of perinatal mortality and disability. Birthweight percentiles form a reference that allows the detection of neonates at higher risk of neonatal and postneonatal morbidity. The aim of the study is to present updated national birthweight percentiles by gestational age for male and female twins born in Australia.

**Methods:**

Population data were extracted from the Australian National Perinatal Data Collection for twins born in Australia between 2001 and 2010. A total of 43,833 women gave birth to 87,666 twins in Australia which were included in the study analysis. Implausible birthweights were excluded using Tukey’s methodology based on the interquartile range. Univariate analysis was used to examine the birthweight percentiles for liveborn twins born between 20 and 42 weeks gestation.

**Results:**

Birthweight percentiles by gestational age were calculated for 85,925 live births (43,153 males and 42,706 females). Of these infants, 53.6 % were born preterm (birth before 37 completed weeks of gestation) while 50.2 % were low birthweight (<2500 g) and 8.7 % were very low birthweight (<1500 g). The mean birthweight decreased from 2462 g in 2001 to 2440 g in 2010 for male twins, compared with 2485 g in 1991–94. For female twins, the mean birthweight decreased from 2375 g in 2001 to 2338 g in 2010, compared with 2382 g in 1991–94.

**Conclusions:**

The birthweight percentiles provide clinicians and researchers with up-to-date population norms of birthweight percentiles for twins in Australia.

## Background

Birthweight remains one of the strongest predictors of perinatal mortality and disability [1, 2]. Birthweight percentiles form a reference that incorporates weight and gestational age of infants at birth and are used as an adjunct for detecting neonates with suspected intra-uterine growth impairment and those at higher risk of neonatal and postneonatal morbidity. Twin births account for about 3 % of all births in Australia but make a significantly greater contribution to perinatal morbidity and mortality than singleton births [3]. Australia’s first birthweight percentiles for twin births based on national population data were published in 1999 using live twins born during 1991–94 [4]. Marked socio-demographic changes in maternal characteristics and clinical practice have occurred during the period since this publication including increased maternal age, reduced smoking rate, and increased usage of assisted reproductive technology [2].

The aim of the study is to present updated national birthweight percentiles for all male and female liveborn twins born in Australia over the 10-year period between 2001 and 2010.

## Methods

Population-based data on twins born in Australia between January 2001 and December 2010 were obtained from Australian Institute of Health and Welfare National Perinatal Data Collection (NPDC). The NPDC is a national collation of jurisdictional population-based cross sectional data collections of pregnancy, childbirth and perinatal outcomes. Information is included in the NPDC on both live births and stillbirths of at least 400 g birthweight or at least 20 weeks gestation.

Records with missing birthweight, infant sex or gestational age values were excluded from calculating the birthweight-by-gestation percentiles. In addition, records with implausible birthweight were identified using Tukey’s methodology [5] based on the interquartile range. Birthweights for each sex and gestational age combination that fell below the first quartile minus twice the interquartile range (lower Tukey limit) or above the third quartile plus twice the interquartile range (higher Tukey limit) were considered outliers and were excluded from the analyses.

Level of remoteness was based on the geographical location of the usual residence of the mother, and was classified into five groups: major cities of Australia, inner regional Australia, outer regional Australia, remote Australia and very remote Australia.

Univariate analysis was used to examine the birthweight distributions and to determine the interquartile range for each gestational age for twins born in Australia. After removing outliers exact percentiles of birthweight in grams were calculated for each gestational week between 20 and 42 weeks. Results for the 5th and 95th percentile are presented only for gestational ages with a minimum of 100 records and the 10th and 90th percentile are plotted only for gestational ages with a minimum of 50 records, to be consistent with previously published Australian birthweight percentiles [2, 4]. Student t-test was used to examine the mean birthweight difference between twins born in 1991–94 and 2010. General linear model was used to investigate the trends for mean birthweight for male and female twins born between 2001 and 2010. All analyses were carried out using SAS for Windows, version 9.3 (SAS Inc, Cary, NC).

Ethics approval for this study was granted by the Human Research Ethics Advisory Panel of the University of New South Wale, Australia (Reference number: 2013-7-07) and Australian Institute of Health and Welfare Ethics Committee (Reference number: EO 2013/2/17). As secondary data analysis of de-identified data set, additional consent from participants was not required. Approval for use of data was provided by all states and territories.

## Results

Between 2001 and 2010, a total of 43,833 women gave birth to 87,666 twins in Australia. Table 1 presents the maternal demographic and obstetric characteristics of these women. 1741 (2.0 %) births (1737 stillbirths and 4 births with unknown vital status at birth) were not considered further.

**Table 1:** Maternal characteristics of women who gave birth to twins, Australia, 2001-2010

Maternal characteristics	Number & percentage
Total	43,833 (100.0 %)
Maternal age (years)	
< 20	873 (2.0 %)
20-24	4076 (9.3 %)
25-29	10,468 (23.9 %)
30-34	15,944 (36.4 %)
35-39	10,412 (23.8 %)
> =40	2055 (4.7 %)
Not stated	5 (0.0 %)
Parity	
Primiparas	17,971 (43.3 %)
Multiparas	23,466 (56.6 %)
Not stated	37 (0.1 %)
Country of birth	
Australia	31,682 (72.3 %)
Overseas	11,944 (27.2 %)
Not stated	207 (0.5 %)
Smoking during pregnancy	
Yes	4271 (13.9 %)
No	25,968 (84.7 %)
Not stated	420 (1.4 %)
Remoteness	
Major Cities	30,579 (69.8 %)
Inner Regional	8136 (18.6 %)
Outer Regional	3959 (9.0 %)
Remote	738 (1.7 %)
Very remote	367 (0.8 %)
Not stated	17 (0.0 %)

Among the 85,925 live births (43,153 males and 42,706 females), 53.6 % were born preterm (birth before 37 completed weeks of gestation) while 50.2 % were low birthweight (<2500 g) and 8.7 % were very low birthweight (<1500 g) (Table 2). More than half of liveborn twins were admitted to a special care nursery or neonatal intensive care unit (58.6 %) or required some type of active resuscitation measures (54.0 %) (Table 2). The median length of stay in hospital for twins was 6 days (interquartile range: 5 – 13 days). The 121 (0.1 %) records missing one or more of the key variables (sex, birthweight and gestational age), and 134 (0.2 %) lower Tukey limit and 207 (0.2 %) higher Tukey limit outliers were excluded. Percentiles were calculated for the remaining 85,436 infants.

**Table 2:** Live twin births, Australia, 2001-2010

Infant characteristics	Number & percentage
Total	85,925 (100.0 %)
Sex	
Male	43,153 (50.2 %)
Female	42,706 (49.7 %)
Not stated	66 (0.1 %)
Birthweight (g)	
< 1500	7461 (8.7 %)
1500-2499	35,689 (41.5 %)
2500-2999	30,403 (35.4 %)
3000-3999	12,262 (14.3 %)
> =4000	68 (0.1 %)
Not stated	42 (0.0 %)
Gestational age (weeks)	
20-27	2845 (3.3 %)
28-31	5501 (6.4 %)
32-36	37,745 (43.9 %)
37-41	39,792 (46.3 %)
> =42	26 (0.0 %)
Not stated	16 (0.0 %)
Presentation	
Vertex	58,727 (68.3 %)
Breech	23,605 (27.5 %)
Other	2566 (3.0 %)
Not stated	1027 (1.2 %)
Apgar score at 5 minutes	
0-3	1124 (1.3 %)
4-6	1810 (2.1 %)
7-10	82,849 (96.4 %)
Not stated	142 (0.2 %)
Resuscitation	
Yes	46,427 (54.0 %)
No	34,581 (40.2 %)
Not stated	4917 (5.7 %)
Admission to NICU	
Yes	47,674 (58.6 %)
No	33,370 (41.0 %)
Not stated	278 (0.3 %)
Length of stay	
Less than 1 day	2645 (3.1 %)
1 day	1502 (1.7 %)
2 day	2582 (3.0 %)
3 day	5102 (5.9 %)
4 day	9289 (10.8 %)
5 day	12,231 (14.2 %)
6 day	9704 (11.3 %)
7-13 days	21,520 (25.0 %)
14-20 days	9138 (10.6 %)
21-27 days	4858 (5.7 %)
28 or more days	7204 (8.4 %)
Not stated	150 (0.2 %)

**Fig. 1:**
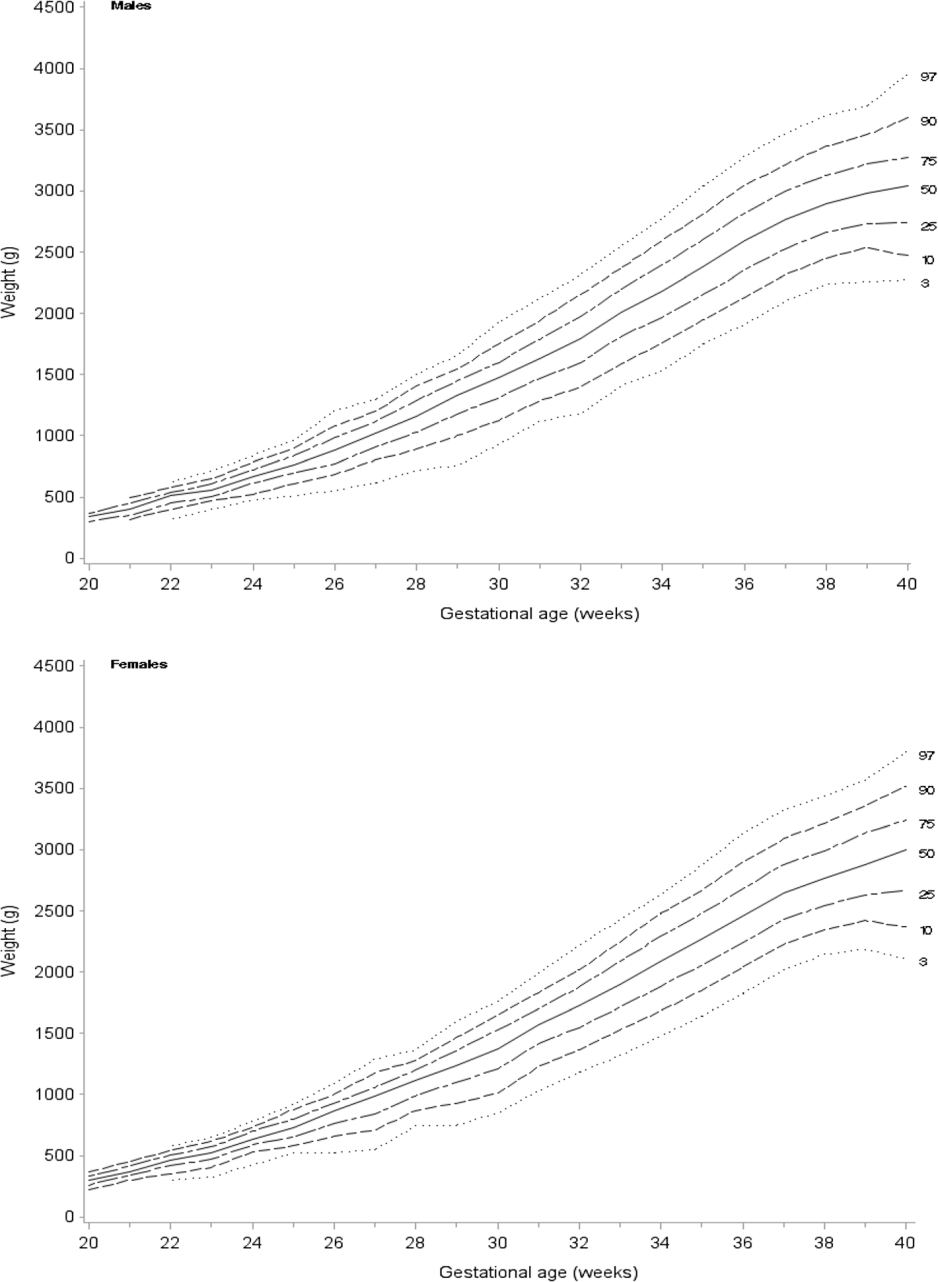
Birthweight percentiles for liveborn twins, by sex, Australia, 2001–2010

**Fig. 2:**
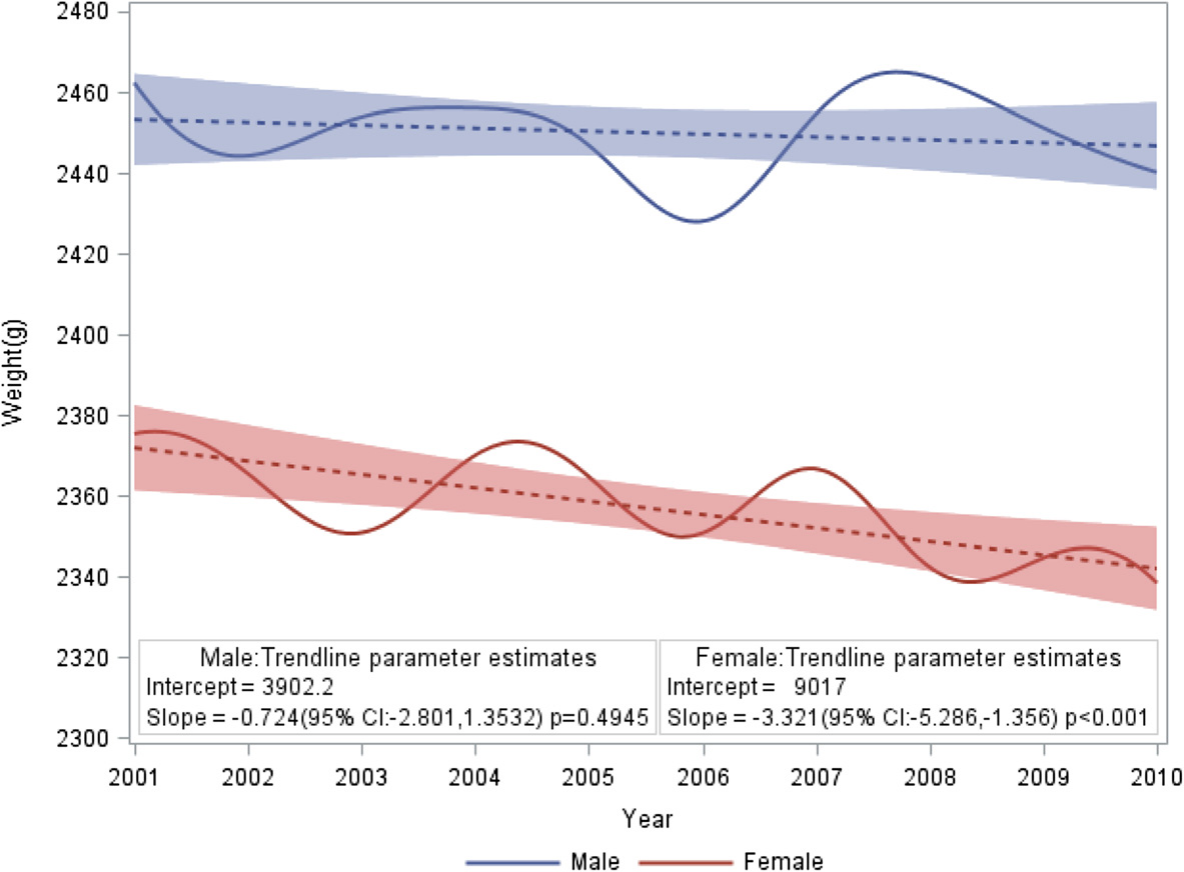
Mean birthweight of liveborn twins, by sex, Australia, 2001–2010

Figure 1 shows birthweight percentiles by gestational age for liveborn twins by infant sex; exact birthweight percentiles are shown in Table 3 and Table 4. The mean birthweight slightly decreased from 2462 g in 2001 to 2440 g in 2010 for male twins (*p* = 0.49). For female twins, the mean birthweight significantly decreased from 2375 g in 2001 to 2338 g in 2010 (*p* < 0.001) (Fig. 2). Compared with twins born in Australian 1991–94, the mean birthweight was significantly lower for twins born in 2010 for both male (2485 g versus 2440 g, *p* < 0.001) and female (2382 g versus 2338 g, *p* < 0.001).

**Table 3:** Birthweight percentiles for male liveborn twins, Australia, 2001-2010

Gestation (weeks)	No. of births	Mean (g)	Standard deviation	Birthweight percentile (g)								
				p3	p5	p10	p25	p50	p75	p90	p95	p97
20	42	338	50	.	.	.	300	340	369	.	.	.
21	90	402	73	.	.	320	355	400	450	495	.	.
22	131	495	72	320	350	400	450	510	540	580	600	620
23	166	560	77	400	440	470	505	560	610	650	695	718
24	216	666	96	477	490	525	617	673	724	784	820	840
25	234	760	114	510	540	610	695	760	840	900	940	968
26	297	883	161	555	610	686	775	880	986	1080	1156	1205
27	323	1010	161	616	716	804	912	1019	1116	1200	1270	1300
28	478	1153	201	720	780	890	1030	1160	1290	1410	1450	1505
29	563	1297	219	757	875	1000	1180	1327	1450	1545	1625	1660
30	720	1453	246	930	991	1128	1314	1477	1600	1755	1850	1928
31	1059	1628	255	1120	1172	1285	1470	1632	1790	1942	2060	2120
32	1638	1784	292	1180	1275	1400	1596	1799	1972	2155	2245	2316
33	2040	1999	304	1410	1469	1590	1815	2010	2200	2370	2486	2555
34	3408	2179	328	1530	1618	1760	1970	2180	2400	2600	2704	2780
35	4503	2382	340	1755	1830	1950	2155	2380	2600	2810	2950	3040
36	7319	2589	359	1910	1995	2135	2350	2595	2820	3044	3185	3280
37	10665	2768	355	2100	2190	2315	2530	2765	3000	3215	3356	3470
38	7601	2901	357	2240	2320	2450	2665	2894	3130	3365	3510	3620
39	1129	2982	364	2260	2415	2538	2730	2980	3220	3460	3600	3695
40	308	3031	427	2275	2330	2470	2743	3040	3275	3600	3790	3960
41	25	3199	558	.	.	.	2805	3015	3500	.	.	.
42	8	2969	459	.	.	.	2500	2980	3400	.	.	.

**Table 4:** Birthweight percentiles for female liveborn twins, Australia, 2001-2010

Gestation (weeks)	No. of births	Mean (g)	Standard deviation	Birthweight percentile (g)								
				p3	p5	p10	p25	p50	p75	p90	p95	p97
20	49	299	57	.	.	.	260	296	330	.	.	.
21	94	374	58	.	.	300	340	370	415	450	.	.
22	107	459	75	300	330	350	420	460	508	545	570	580
23	143	517	83	325	350	405	470	520	570	620	640	650
24	176	635	87	425	480	530	589	638	700	731	751	780
25	167	728	113	520	547	580	656	730	800	878	907	920
26	241	845	141	525	580	660	760	864	930	1000	1052	1090
27	308	951	186	550	598	710	840	983	1060	1180	1240	1290
28	384	1090	169	745	795	870	991	1112	1200	1280	1345	1365
29	542	1219	211	750	808	930	1101	1235	1360	1465	1536	1601
30	697	1355	244	850	896	1010	1210	1375	1530	1655	1725	1760
31	990	1551	239	1030	1105	1227	1415	1568	1700	1833	1930	1990
32	1618	1713	262	1182	1248	1365	1550	1730	1880	2020	2140	2220
33	2037	1895	288	1320	1400	1530	1713	1900	2086	2250	2360	2430
34	3322	2085	308	1482	1555	1685	1885	2090	2292	2480	2570	2635
35	4558	2267	324	1640	1715	1850	2060	2270	2480	2670	2790	2875
36	7145	2464	339	1825	1905	2035	2240	2460	2680	2900	3030	3130
37	10695	2656	340	2020	2100	2225	2430	2650	2880	3090	3230	3326
38	7720	2774	339	2145	2230	2349	2545	2765	2990	3215	3350	3435
39	1154	2884	373	2185	2280	2420	2630	2875	3140	3360	3500	3570
40	320	2958	438	2110	2180	2368	2668	3000	3240	3518	3653	3800
41	22	2976	322	.	.	.	2795	2933	3220	.	.	.
42	11	3001	497	.	.	.	2540	2910	3440	.	.	.

## Discussion

We developed the contemporary population-based birthweight percentile charts to provide an up-to-date reference for twins born in Australia.

Growth in twin pregnancies slows progressively from around 32 weeks until term [6]. When comparing the dataset in this study with a singleton population from a similar time period [2] this phenomenon is confirmed. The median gestation-specific birthweight for twins were remarkably similar to those for singletons from a similar era until around 32 weeks gestation. The difference then becomes progressively more pronounced, reaching 640 g for males and 480 g for females at 40 weeks. It has been postulated that this slowing of growth late in a multiple pregnancy is a physiologically adaptive process that favours developmental maturity at the expense of fetal size [6]. However, there is a limit to this physiological adaptation and eventually the growth restriction becomes a pathological process. This is evidenced by studies consistently highlighting the risks of late fetal death in twins, particularly in relation to growth restriction [7–9].

Twin pregnancies have an increased risk of adverse outcome compared to single pregnancies. The increase in perinatal mortality and morbidity is primarily related to preterm delivery, complications of monochorionicity, and fetal growth restriction. Accurate population data is required to accurately diagnose growth restriction in twin pregnancies.

Secular trends for a decrease in the overall mean birthweight for liveborn twins were observed for both male and females in Australia in the study period. In contrast the mean birthweight for liveborn singletons in Australia from a similar time period has been relatively stable [2]. The observed decline in the mean birthweight for twins over time was partially explained by the downward shift in the distribution of gestational age. The mean gestational age was 35.4 weeks for twins in 2001, compared with 35.1 weeks in 2010 [10, 11]. Preterm birth occurred in 51.2 % of twins in 2001 and 56.7 % in 2010 [10, 11]. This decreased mean gestational age and increased pre-term births rate among twins might be attributed to improved intrapartum survival at earlier gestations, earlier obstetric interventions and increased usage of assisted reproductive technology [12, 13].

Population-based birthweight percentile charts for twins are scarce [14–17]. When comparing Australian birthweight percentile charts with others, significant differences in birth size are observed between populations. At 40 weeks gestation, the mean birthweight of Australian live born twins are markedly lower than Norwegian and Finnish counterparts [15, 17] but the median birthweight of Australian twins are heavier than Canadian and Japanese twins [14, 16]. Sankilampi et al. has stated that the differences in term birth size is largely associated with differences in genetic background rather than maternal nutrition or healthcare as this is comparable between these developed countries [17].

There is inherent appeal in the customisation of growth charts that incorporate maternal weight, height, ethnicity as well as plurality. The intent is to identify fetuses that are small as a consequence of growth restriction rather than constitutionally small for clinical decision-making. Several large observational studies have suggested that customised charts improve the identification of infants with intrauterine growth restriction compared with population based charts, although contrasting opinions exist [18–22]. Birthweight centiles provide a population reference describing fetal growth in the population rather than a standard for assessing individual growth [23]. They also provide the basis for characterising newborn size for longitudinal studies of childhood outcomes. The usefulness of customised birthweight percentiles has been debated. The value of plurality-specific centiles has been established but the literature remains divided on the benefits of customisation for other characteristics and the need for multiple reference charts [24]. Maternal height and weight have been included in the NPDC from 2010, but agreed national standards have not yet been implemented. Further studies are required to examine whether customised growth charts adjusted for maternal size and ethnicity contributes to improved prediction of adverse perinatal outcomes.

The provision of these updated birthweight percentile charts allows clinicians and researchers to re-evaluate the success of pregnancy management by determining the rate of detection of growth restriction. As previously described, growth restriction is the major cause of late fetal death in twin pregnancies and its accurate diagnosis a focal point of good obstetric care.

One limitation of this study is that birthweight percentile charts do not measure intrauterine growth but rather size at birth. The birthweight of babies born prematurely is likely to be influenced by the pathological process leading to preterm birth and therefore likely to differ from those remaining *in utero* until term [25, 26]. It has been argued that the preterm births should be assessed using estimated fetal weight rather than birthweight percentile charts as preterm neonates are disproportionately affected by the fetal growth restriction [25]. However, the accuracy of estimated fetal weight is limited by the ability of obtaining accurate measurements included within the computation of estimated fetal weight and the formula used for computation [26, 27]. To date, there has been no Australian chart published for sonographic standards for estimated fetal weight and the Australasian Society for Ultrasound in Medicine’s position is that ‘No formula for estimating fetal weight has achieved an accuracy which enables us to recommend its use’ [27, 28]. In such cases, the population-based birthweight percentile charts presented in this study provide a valuable reference for clinicians and researchers assessing the prognosis of twins in Australia.

## Conclusions

This study presents the up-to-date national population-based birthweight percentile charts for male and female live born twins in Australia. These new charts provide a valuable reference for clinicians and researchers correctly identifying high-risk twins in Australia.

## Abbreviations

AIHW: Australian Institute of Health and Welfare
NPDC: National Perinatal Data Collection

## References

[1] Goldenberg RL, Culhane JF (2007). Low birth weight in the United States. Am J Clin Nutr.

[2] Dobbins TA, Sullivan EA, Roberts CL, Simpson JM (2012). Australian national birthweight percentiles by sex and gestational age, 1998–2007. Med J Aust.

[3] Li Z, Zeki R, Hilder L, Sullivan EA (2013). Australia’s mothers and babies 2011.

[4] Roberts CL, Lancaster PA (1999). National birthweight percentiles by gestational age for twins born in Australia. J Paediatr Child Health.

[5] Tukey JW (1977). Exploratory data analysis.

[6] Blickstein I, Blickstein I, Keith LG (2005). Intrauterine growth. Multiple pregnancy: epidemiology, gestation & perinatal outcome.

[7] Dodd JM, Crowther CA, Haslam RR, Robinson JS (2012). Elective birth at 37 weeks of gestation versus standard care for women with an uncomplicated twin pregnancy at term: the twins timing of birth randomised trial. BJOG.

[8] Hack KE, Derks JB, Elias SG, Franx A, Roos EJ, Voerman SK (2008). Increased perinatal mortality and morbidity in monochorionic versus dichorionic twin pregnancies: clinical implications of a large Dutch cohort study. BJOG.

[9] Page JM, Pilliod RA, Snowden JM, Caughey AB (2015). The risk of stillbirth and infant death by each additional week of expectant management in twin pregnancies. Am J Obstet Gynecol.

[10] Laws PJ, Sullivan EA (2004). Australia’s mothers and babies 2001. AIHW Cat No. PER 25.

[11] Li Z, Zeki R, Hilder L, Sullivan E (2012). Australia’s mothers and babies 2010. Canberra: Perinatal statistics series no. 27. Cat. no. PER 57.

[12] Nassar N, Schiff M, Roberts CL (2013). Trends in the distribution of gestational age and contribution of planned births in New South Wales, Australia. PLoS One.

[13] Verstraelen H, Goetgeluk S, Derom C, Vansteelandt S, Derom R, Goetghebeur E (2005). Preterm birth in twins after subfertility treatment: population based cohort study. BMJ.

[14] Arbuckle TE, Wilkins R, Sherman GJ (1993). Birth weight percentiles by gestational age in Canada. Obstet Gynecol.

[15] Glinianaia SV, Skjaerven R, Magnus P (2000). Birthweight percentiles by gestational age in multiple births. A population-based study of Norwegian twins and triplets. Acta Obstet Gynecol Scand.

[16] Kato N (2004). Reference birthweight range for multiple birth neonates in Japan. BMC Pregnancy Childbirth.

[17] Sankilampi U, Hannila ML, Saari A, Gissler M, Dunkel L (2013). New population-based references for birth weight, length, and head circumference in singletons and twins from 23 to 43 gestation weeks. Ann Med.

[18] Figueras F, Figueras J, Meler E, Eixarch E, Coll O, Gratacos E (2007). Customised birthweight standards accurately predict perinatal morbidity. Arch Dis Child Fetal Neonatal Ed.

[19] Hutcheon JA, Zhang X, Cnattingius S, Kramer MS, Platt RW (2008). Customised birthweight percentiles: does adjusting for maternal characteristics matter?. BJOG.

[20] Hutcheon JA, Zhang X, Platt RW, Cnattingius S, Kramer MS (2011). The case against customised birthweight standards. Paediatr Perinat Epidemiol.

[21] Larkin JC, Hill LM, Speer PD, Simhan HN (2012). Risk of morbid perinatal outcomes in small-for-gestational-age pregnancies: customized compared with conventional standards of fetal growth. Obstet Gynecol.

[22] Mongelli M, Figueras F, Francis A, Gardosi J (2007). A customized birthweight centile calculator developed for an Australian population. Aust N Z J Obstet Gynaecol.

[23] Bertino E, Milani S, Fabris C, De Curtis M (2007). Neonatal anthropometric charts: what they are, what they are not. Arch Dis Child Fetal Neonatal Ed.

[24] Joseph KS, Fahey J, Platt RW, Liston RM, Lee SK, Sauve R (2009). An outcome-based approach for the creation of fetal growth standards: do singletons and twins need separate standards?. Am J Epidemiol.

[25] Cooke RW (2007). Conventional birth weight standards obscure fetal growth restriction in preterm infants. Arch Dis Child Fetal Neonatal Ed.

[26] Ehrenkranz RA (2007). Estimated fetal weights versus birth weights: should the reference intrauterine growth curves based on birth weights be retired?. Arch Dis Child Fetal Neonatal Ed.

[27] Hui L (2008). Australian charts for assessing fetal growth: a review. ASUM Ultrasound Bulletin.

[28] Australasian Society for Ultrasound in Medicine (2001). Statement on normal ultrasonic fetal measurements.

